# The microalga *Volvox carteri* as a cell supportive building block for tissue engineering

**DOI:** 10.1016/j.mtbio.2024.101013

**Published:** 2024-02-29

**Authors:** Mathilde Stricher, Pascale Vigneron, Frederic Delbecq, Claude-Olivier Sarde, Christophe Egles

**Affiliations:** a Université de Technologie de Compiègne, CNRS, Biomechanics and Bioengineering, Centre de Recherche Royallieu, CEDEX CS 60 319, 60 203, Compiègne, France; b Université de Technologie de Compiègne, ESCOM, TIMR (Integrated Transformations of Renewable Matter), Centre de Recherche Royallieu, CEDEX CS 60 319, 60 203, Compiègne, France; c Univ Rouen Normandie, INSA Rouen Normandie, CNRS, Normandie Univ, PBS UMR 6270, 55 Rue Saint-Germain, 27 000, Évreux, France

**Keywords:** Algal extracellular matrix, Vegetal alternative, Modular self-assembly tissue engineering, Soft tissue augmentation, Adipogenesis

## Abstract

**Background:**

*V. carteri f. nagariensis* constitutes, in its most simplified form, a cellularized spheroid built around and stabilised by a form of primitive extracellular matrix (ECM).

**Methods:**

We developed a modular approach to soft tissue engineering, by compact stacking V. *carteri*-based building blocks. This approach is made possible by the structure and cell adhesive properties of these building blocks, which results from the composition of their algal ECM.

**Results:**

A primary biocompatibility assessment demonstrated the cytocompatibility of the algal suspension, its histogenesis-promoting properties, and that it did not induce an inflammatory response *in vitro*. These results allowed us to consider the use of this algal suspension for soft tissue augmentation, and to initiate an *in vivo* biocompatibility study. *V. carteri* exhibited cellular fate-directing properties, causing (i) fibroblasts to take on an alkaline phosphatase^+^ stem-cell-like phenotype and (ii) both human adipose-derived stem cells and mouse embryonic stem cells to differentiate into preadipocytes to adipocytes. The ability of *V. carteri* to support histogenesis and adipogenesis was also observed *in vivo* by subcutaneous tissue augmentation of athymic mice, highlighting the potential of *V. carteri* to support or influence tissue regeneration.

**Conclusions:**

We present for the first time *V. carteri* as an innovative and inspiring biomaterial for tissue engineering and soft tissue regeneration. Its strategies in terms of shape, structure and composition can be central in the design of a new generation of bio-inspired heterogeneous biomaterials recapitulating more appropriately the complexity of body tissues when guiding their regeneration.

## Graphical abstract

**Figure:**
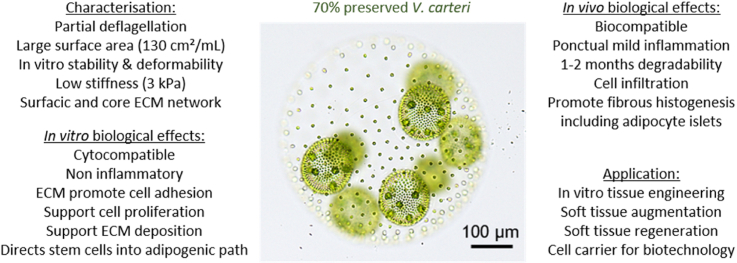

## 1 Background

Over the past years, tissue engineering strategies have gradually shifted away from the conventional top-down approach of scaffold cellularization to a more malleable modular design based on coupled individual living building blocks (LBB). In this approach, LBBs of varying complexity are combined via self, directed, or remote assembly in various dimensions and geometries (e.g., cell fibers, cell sheets, and spheroids), optionally including biomaterials and biomolecules [1]. This design allows for a more facilitated and controlled introduction of progressive heterogeneity (e.g., zonal or gradient seedings and substrate transitions) and structural complexity (e.g., vascular or neuronal networks and specific microstructures) that more accurately mimic true *in vivo* tissue environments.

The modular approach is thus founded on the notion that progressive multicellularity efficiently promotes the emergence of a complex organization. This concept is widely shared in the evolutionary community, as the acquisition of multicellularity constitutes one of the major transitions toward complexity in evolution [2]. Although transitions from single-celled organisms to multicellular ones have occurred multiple times independently throughout evolution, the one carried out by the green algae volvocine has been one of the most studied and is considered like a remarkable model to decipher the genetic bases of multicellularity and cell differentiation [3].

Indeed, volvocine algae represent a unique evolutionary continuum in the acquisition of multicellularity, which extends from the unicellular organism *Chlamydomonas reinhardtii* to the multicellular alga *Volvox carteri,* which represents its most advanced manifestation. Their phylogenomic analysis, initiated by Kirk [4], defines a progressive acquisition of fundamental processes (e.g., cell-cell adhesion, organism polarity, extracellular matrix (ECM) expansion, and cell differentiation mechanisms) that have been widely agreed upon and continuously refined ever since [5,6].

V. *carteri*, like other green algae, can be found worldwide, particularly in low-turbulent, low-turbidity, high-alkalinity, and nutrient-rich freshwater ecosystems [7,8]. An adult spheroid is a 100–500 μm algal ECM-based sphere comprising an outer sheet, consisting of a monolayer of ∼2000–4000 equidistant biflagellate somatic cells whose general appearance is reminiscent of their distant ancestor *Chlamydomonas*, and enclosing ∼16 much larger reproductive aflagellated gonidia cells.

During its growth, each alga produces a considerable amount of ECM, accounting for up to 99% of its total volume and forming a continuum of zones that seem defined by specific molecular compositions [9]. The algal ECM, however, is primarily composed of negatively charged fibrous hydroxyproline-rich glycoproteins (HRGP), mainly Volvocale-specific glycoproteins termed “pherophorins” which may be considered functionally analogous to animal collagen [10,11].

In a primary biomimicry and bioinspiration initiative driven by the necessity to develop plant alternatives to animal-derived products for tissue engineering, we have studied the potential of *V. carteri* to respond to this challenge. After ascertaining its overall biocompatibility, we developed a modular approach to soft tissue engineering based on compact stacking using animal cell-seeded *V. carteri f. nagariensis* spheroids as living building blocks. We demonstrated that this microalga displays a cell-adhesive glycoprotein-based scaffold that supports histogenesis and can direct adipogenesis both *in vitro* and *in vivo*.

## 2 Methods

Unless otherwise stated, culture reagents were purchased from Gibco™ (ThermoFisher Scientific, Waltham, MA, USA), primary cells and lineages were provided by ATCC (Manassas, VA, USA), and cultured in 4.5 g/L glucose Dulbecco's modified eagle's medium (DMEM), supplemented with 10% V/V decomplemented Fetal Bovine Serum (FBS), 100 U/mL penicillin, 100 μg/mL streptomycin, and 2 mM of L-Glu. Characterized FBS (HyClone, Logan, UT, USA) was used for primary neonatal Human Dermal Fibroblasts (HDFn) culture. Human Umbilical Vein Endothelial Cells (HUVEC) were cultured in M199 medium, 10% V/V FBS, 50 μg/mL of heparin B, 2 mM of L-Glu, 100 units/ml of penicillin, and 100 μg/mL of streptomycin. Human adipose-derived mesenchymal stem cells (hASC) were maintained in Mesenchymal Stem Cell (MSC) basal medium enriched with MSC supplement (ATCC, Manassas, VA, USA), 100 units/mL of penicillin, and 100 μg/mL of streptomycin.

### 2.1 *V. carteri* culture and processing

V. *carteri f. nagariensis* (NIES397) axenic strain was provided by NIES based in Tsukuba, Japan. This algal strain was grown continuously in a 300-mL batch culture under sterile conditions in VT medium pH 7.5 at 25 °C under an alternating 14:10 h day-night cycle in a growth chamber fitted with a 15,000-lux light system (POL-EKO-APARATURA, Wodzisaw Sski, Poland) [12]. Spheroids were harvested by filtration using a 100 μm porosity sieve (Haver & Boecker oHG, Oelde, Germany). The algal suspension was either freshly used or preserved with 70% ethanol or 4% paraformaldehyde (PFA). *V. carteri* extracts were produced by crushing with a UP400S ultrasonic device (Hielscher Ultrasonics, Teltow, Germany) using 0.5-s cycles at 400 W and 24 kHz for 1 min. After 1 min of 3500 Relative Centrifugal Force (RCF) centrifugation, the soluble fraction was collected and filtered at 0.8 μm.

### 2.2 Investigation of *V*. *carteri* adequacy as a substrate for *in vitro* cell culture

*Cytotoxicity investigation* was adapted from the ISO 10993-5 standards*:* A first testing extract was prepared by incubating rinsed, 70% ethanol-fixed spheroids for 24 h at 37 °C with agitation in a 1:1 algal pellet:culture medium ratio. The second extract of a fresh *V*. *carteri* spheroids pellet was produced in culture medium and ultrasonically crushed on ice as previously described. L929 mouse fibroblast (ATCC-CC1-1) monolayers pre-cultured in 96-well plates for 24 h at 37 °C with 5% CO_2_ were treated for an additional 24 h with 100 μL of testing extract. L929 mitochondrial activity was quantified after a 2-hr incubation in 3-(4,5-dimethylthiazol-2-yl)-5-(3-carboxymethoxyphenyl)-2-(4-sulfophenyl)-2H-tetrazolium (MTS) solution (1:5; Promega, Madison, WI, USA). Cellular viability was determined as the 492 nm-absorbance ratio of the extract to the positive control (culture medium only). A material inducing more than 70% cellular viability was deemed non-toxic.

In *vitro inflammation analysis*: J744 macrophages (ECACC-85011428) were seeded at a density of 60,000 cells/cm^2^ in 12-well plates and incubated overnight. Negative and positive control conditions were generated through exposure to culture medium alone or containing 20 μg/mL of lipopolysaccharide toxin (LPS). The supernatants cytokines and chemokines concentrations (pg/mL) were quantified using the V-plex Proinflammatory Panel 1, Cytokine Panel 1, and Th17 Panel 1 kits from Meso Scale Diagnostics (MSD, MD, USA) according to the manufacturer's instructions. The concentrations were expressed as a log2-fold change from the untreated control. The data were processed under principal component analysis following an autoscaling normalization using MetaboAnalyst 5.0 Server (https://www.metaboanalyst.ca/).

*α-D-mannosyl and α-D-glucosyl-containing glycans and glycoproteins Concanavalin A (ConA) lectin staining:* 4% PFA-fixed *V. carteri* spheroids were prepared for staining by performing two 10-min PBS washes, a 10-min 5% V/V Triton X-100 permeabilization, a 5-min PBS wash, and a 15-min 3% w/v Bovine Serum Albumin (BSA) non-specific site saturation. Concanavalin A Alexa Fluor™ 488 (ConA AF 488, Invitrogen™, Waltham, MA, USA) labelling was then pursued according to the manufacturer's instructions and observed using confocal microscopy (Zeiss LSM 710, Zeiss, Jena, Germany).

*Estimation of the cell adhesive properties of V. carteri:* non-adhesive 6-well plates (Evergreen Scientifics, Vernon, CA, USA) were precoated with 1 mL of *V. carteri* extract per well for 1 h at 37 °C. The cell suspensions of HDFn (C-004-5C) and HUVEC (CRL-1730TM) was then seeded at a density of 5000 cells/cm^2^ on a non-adhesive or algal extract pre-coated surface. Cell confluency was evaluated at 24 and 48 h on contrast-phase light microscopic acquisitions using Fiji (https://fiji.sc/) and the phase contrast microscopy segmentation toolbox (PHANTAST) for Fiji plugin.

*Estimation of V. carteri spheroids in vitro stability and deformability:* 500 μL of algal suspension containing approximately 7500 spheroids was placed in 12-well culture inserts (353,181, Falcon™, Corning, NY, USA). The deformability of the algae was monitored by phase-contrast microscopy imaging on days 2, 7, 14, and 21. The circularity of each colony was determined using Fiji and calculated as follows:

(1.1) $$Circularity=4\pi \times \frac{Area}{{Perimeter}^{2}}$$

A perfectly circular colony would have a circularity index of 1.

*Nanoindentation test:* Nanoindentation assays were performed using a PIUMA CHIARO nanoindentation system (Optics11, Amsterdam, The Netherlands) on rehydrated Ethanol 70% preserved *V. carteri*. Before testing, calibration of the cantilever sensitivity was performed by indenting the hard surface of a Petri dish. The load generated upon a 15,000-nm indentation at a speed of 3 nm/s was quantified using a 28 μm diameter colloidal probe arranged on a rigid 0.48 N/m cantilever.

### 2.3 Identification of potential ECM components in the predicted *V. carteri* proteome

V. *carteri* protein sequences were identified and retrieved when necessary from Phytozome, the Plant Comparative Genomics portal of the Department of Energy's Joint Genome Institute (Lawrence Berkeley National Laboratory, Berkeley, CA, USA) [updated 2023 Dec 18; accessed 2024 Feb 5]. Retrieved sequences were analyzed in pairwise comparison or against non-redundant protein sequences (nr) database using the blastp algorithm (https://blast.ncbi.nlm.nih.gov/). The amino acid compositions were obtained with the ProtParam tool (https://web.expasy.org/protparam/). Domains, families and functional sites within protein sequences were confirmed whenever possible with the Prosite tool (https://prosite.expasy.org/).

### 2.4 Macrotissue formation using the compact stacking of seeded *V. carteri*-based living building blocks

The seeded *V. carteri*-based LBBs and subsequent macrotissue formation process were standardized for the following cell types: L929, HDFn, HUVEC, C3H10 (ATCC-CCL-226), and ASC (ATCC SCRC-4000).

*Formation of seeded V. carteri-based living building blocks (LBBs):* The algae were primed overnight (ON) with the culture medium associated with the cell type to be seeded. *V. carteri*-based LBBs were formed by mixing a cell suspension of 2 × 10^6^ cells/mL with an algae suspension of 15,000 spheroids/mL at a 1:1 vol ratio, incubating the mix for 45 min in a 15-mL tube, and then for 4 h on a non-cell adhesive petri dish (Greiner Bio-One, Frickenhausen, Germany) or 6-well plate (Evergreen Scientifics, Vernon, CA, USA).

*Self-assembly of seeded V. carteri-based living building blocks into macrotissue:* 1 mL of V. *carteri*-based LBB suspension was then cultured in 12-well plates on culture inserts with a pore size of 3 μm. (353,181, Falcon™, Corning, NY, USA), except for the L929 lineage, which required culture inserts with a 0.4 μm pore size (353,180, Falcon™, Corning, NY, USA). Culture medium was changed every 2–3 days until 21 days of culture. The samples were either processed immediately or fixed with 4% PFA for 1 h at 37 °C.

### 2.5 Characterization of seeded *V. carteri*-based living building blocks and macrotissue

*Cellular activity monitoring:* At days 2, 7, 14, and 21, cellular proliferation was monitored by quantifying HDFn mitochondrial activity using MTS testing. Similarly, the alkaline activity of HDFn phosphatase was measured by contrast-phase microscopy after 1 h of incubation in 1 mL of BCIP®/NBT Liquid Substrate System (Sigma-Aldrich, St. Louis, MO, USA) at 37 °C, in the dark.

*Histology:* Classical sample preparation, hematoxylin-eosin (HE), and optional safran (HES) staining procedures were applied. To better distinguish the contours of the algal spheroids in the formed macrotissue, before cell seeding, the surface of the V. *carteri* spheroids was coupled with rhodamine-NHS (Life Technologies, Carlsbad, CA, USA) according to the manufacturer's instructions. Human cells were counterstained with DAPI and observed via epifluorescence microscopy (Leica Microsystems, Wetzlar, Germany). Adipogenic differentiation was produced with either adipose-derived or C3H10 embryonic stem cells, staining eventual lipid droplets using Oil Red O. After 1 h of 4% PFA fixation at 37 °C, the sample was rinsed twice before incubating ON at RT with 1 mL of 1.5 mg/mL Oil Red O working solution. For confocal microscopy observation, the samples were counterstained with a 1 μg/mL DAPI solution at RT and washed with PBS for 15 min. The macrotissues were then prepared for Phalloidin-iFluor 488 ON staining (1:1000, Abcam, Cambridge, UK) and DAPI counterstaining. The sample was isolated from the insert membrane and transferred to a standard microscope slide or an imaging chamber coverslip (Ibidi, Gräfelfing, Germany) for observation.

### 2.6 Implantation in a model of athymic mice

All experiments were realized in compliance with European Directive 2010-EU63 and the ARRIVE guidelines. The study design, the sample size, the outcomes, and the experimental procedures were approved by the “Comité Régional d’Ethique en Matière d’Expérimentation Animale de Picardie” (CREMEAP; C2EA-96). The chosen experimental animals were pathogen-free 5-week-old male athymic mice (Rj: NMRI-Foxn1 nu/nu, JANVIER LABS, Le Genest-Saint-Isle, France; 30 g). Animals were housed in a temperature- and humidity-controlled room and had food and water *ad libitum*. Both 70% ethanol-preserved *V. carteri* saturated suspension and HDFn-seeded *V. carteri* LBB-based macrotissue samples were implanted subcutaneously. The animals were euthanized after one or two months, and their back skin was harvested and processed under classical histology procedures (Althisia, Troyes, France) for an anatomopathological analysis and interpretation by a certified professional.

## 3 Results

### 3.1 Production of *V. carteri* microalgae material on a laboratory scale

**Fig. 1:**
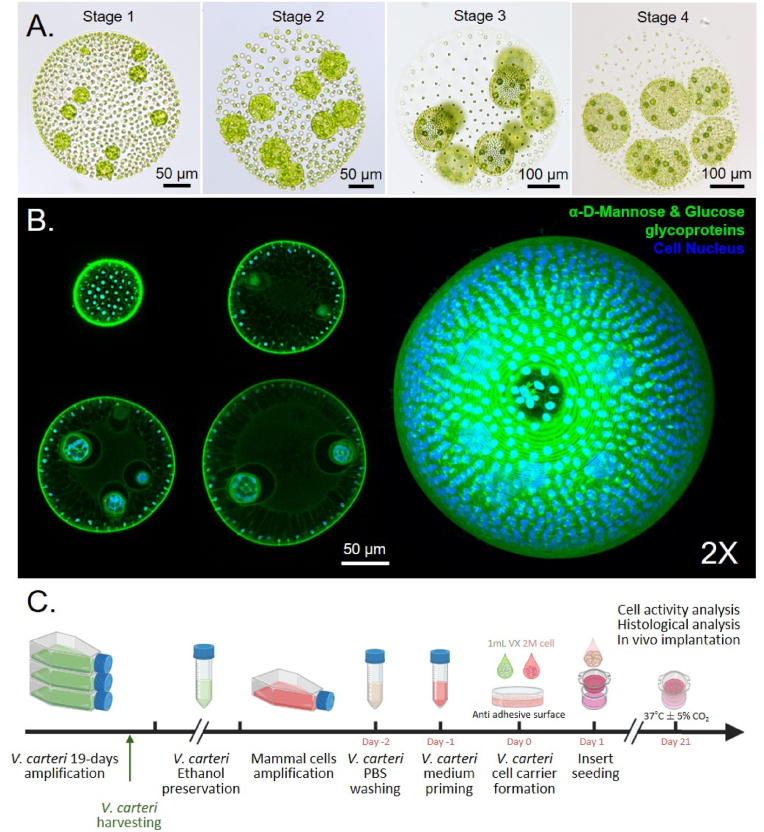
Processing of the algal spherical material to engineer macrotissues. A. Development of a *V. carteri* spheroid from juvenile to adult stage. *V. carteri* juvenile spheroids present ∼2000 biflagellated somatic cells on their surface and up to 16 embryo cells termed as “gonidia” in their core. While the overall diameter of a juvenile spheroid continuously expands by extracellular matrix deposition, its unicellular algal embryo progressively divides symmetrically and then asymmetrically, leading to cell differentiation of somatic cells, which will constitute the external membrane, and gonidia progenitor cells that will grow as future daughter spheroids. Once both the adult and their daughter spheroids reach a critical diameter, the juvenile hatch are discharged; B. Observation of *V. carteri*'s overall structuration and compartmentation established by a highly organized intertwined network of algal glycoproteins. Representative Z-stack and 3D reconstruction of a stage 2 *V. carteri* spheroid labelled for glycoproteins with α-D-Mannose and Glucose sugar groups and DNA content with Concanavalin A (Green) and DAPI; C. Experimental procedure for the formation of *V. carteri* living building blocks-based macrotissues. (For interpretation of the references to color/color in this figure legend, the reader is referred to the Web version of this article.)

*Volvox carteri* was grown axenically in a batch culture system that allowed for the production of 2.52 ± 0.61 million spheroids per liter of medium. This system provided a sufficient, continuous, and regular supply of fresh microalgae material, with maximum production in the growth chamber reaching 151 ± 36.6 million *V. carteri* spheroids per batch. The algal culture being unsynchronized in our culture conditions, the harvested suspension contained algal spheroids at every developmental stage (Fig. 1A), ranging from juvenile spheroids sheltering unicellular gonidia to mature spheroids about to hatch and degenerate upon next generation release. We experienced optimal maintenance of the *V. carteri* batch culture when reseeding and harvesting were carried out on the 19th day of culture, which corresponded to the end of the exponential growth phase. Beyond that point, the culture declined due to medium nutrient depletion, with reduced chlorophyll density, overall colony diameter, the occurrence of mature mother colonies, as well as the increasing appearance of morphologically abnormal colonies (losses of circularity, polarization, and reproductive cells).

The incorporation of the *V. carteri* microalga into mammalian cell cultures to use it as cell support material required preserving their structure as much as possible by fixing, which implied stopping their proliferation, as well as cellular and metabolic activities as quickly as possible to limit any stress response. Histochemical staining with concanavalin A (Fig. 1B) demonstrated the presence of α-d-mannose and α-D-glucose in glycans and glycoproteins inside the cells as well as in the extracellular material. It particularly highlighted the fundamental involvement of the ECM in the composition, formation, and cohesion of the structure of algae. Fixation in 70% ethanol appeared to partially deflagellate the outer somatic cells, permeabilize the colony membrane, releasing small soluble compounds, including chlorophyll pigments, while preserving the overall algal geometry. The 70% ethanol solution was able to preserve the overall integrity and sterility of the alga for up to year (data not shown). It is also a less harmful and more easily eliminated solvent than other fixation methods (e.g., paraformaldehyde). As such, 70% ethanol preservation was preferred for the subsequent formation of *V. carteri* LBB-based macrotissues, as described in Fig. 1C.

The cellular reactivity to the introduction of *V. carteri* as a culture substrate was investigated by analysing cellular activity and dynamics upon direct contact.

### 3.2 In vitro evaluation of V. carteri biocompatibility: cytotoxicity and inflammation

**Fig. 2:**
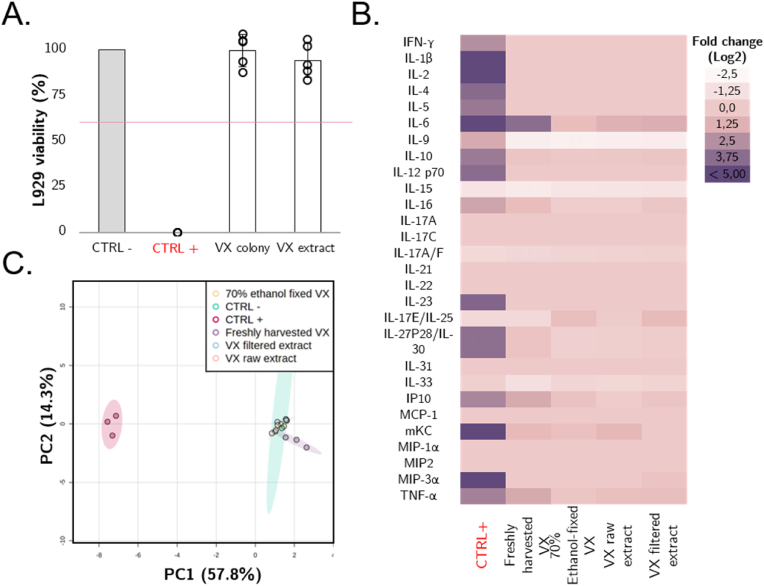
*In vitro* biocompatibility analysis of *V. carteri* material. A. *V. carteri* cytotoxicity evaluation adapted from ISO 10993 standards (N = 5), B. J774.2 murine macrophages inflammatory secretome upon *V. carteri* (VX) exposition. The cytokine and chemokine concentration in the supernatant upon J774.2 macrophage 24 h-exposure was measured by Meso Scale Discovery, expressed as a Log2-fold change of the non-treated control and represented as a heatmap (N = 3). When below or above the detection range, the concentrations were replaced by threshold values. A 2 μg/mL LPS solution was used as a positive control (CTRL+). C. PCA analysis of the *V. carteri*-induced macrophage secretome in regards to both untreated (CTRL-) and treated (CTRL+) controls. The cytokine and chemokine concentration in the supernatant was processed for normalization and PCA analysis with Metaboanalysis software [22].

For *V. carteri*-primed medium (i.e., mimicking the contact with the surface of the alga), a L929 viability of 99.8 ± 8.8% was obtained, while for the sonicated condition (i.e., simulating the full algal compound exposure, both external and internal), a L929 cell viability of 94.2 ± 9.3% was found. Thus, all *V. carteri* conditions demonstrated L929 viability above the 70% threshold. According to ISO 10993 criteria, the algal suspension and extract are considered non-cytotoxic (Fig. 2A).

The murine J774.2 macrophage cytokine secretome heatmap (Fig. 2B) was generated after a 24-h exposure to various *V. carteri* samples in comparison to untreated (CTRL-) and pro-inflammatory LPS-treated (CTRL+) controls, to further assess its biocompatibility. This heatmap highlights the expected acute inflammation induced by 2 μg/mL LPS exposure through the significant secretion of numerous pro-inflammatory cytokines. This pro-inflammatory response was also evidenced via principal component analysis (PCA, Fig. 2C), as the LPS-treated condition represents a cluster isolated from the untreated condition. *V. carteri* samples did not show pro-inflammatory cytokine and chemokine secretions to the extent induced by LPS, as shown by the aggregation of the algal samples around the negative control on the PCA. Only freshly harvested, unfixed *V. carteri* spheroids induced moderate IL-6 and TNF-α secretions.

A PCA was performed without LPS-induced acute inflammation conditions, to more precisely identify variations between algal conditions (Fig. S1). In this PCA, a deviation of the unfixed and freshly harvested spheroids group from the control reference group was observed. This deviation is related to the moderate secretion of pro-inflammatory cytokines and chemokines, TNF-α, IL-6, IP-10, and mKC. Once these algae were fixed with 70% ethanol, we observed a complete re-centring around the control group. A slight variation was also observed for the raw extract condition, corresponding to the slight secretion of IL-6, IL-10, IL-12, mKC, and TNF-α. While algal preservation and extract filtration were beneficial, *V. carteri* proved to be generally non-inflammatory.

### 3.3 Characterization of V. carteri living building blocks’ compact stacking in saturated suspension

**Fig. 3:**
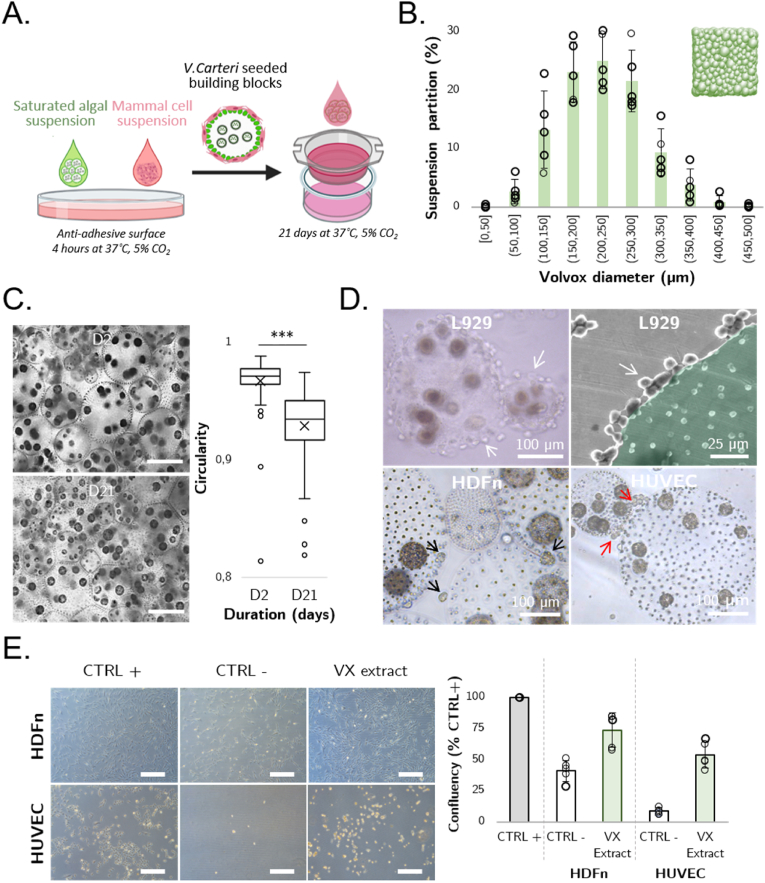
Production of compactable and deformable seeded *V. carteri* living building blocks. A. Living building blocks formation principle, B. Normal distribution of *V. carteri* spheroid diameter in the saturated algal suspension (N = 5; Shapiro-Francia Test; W' = 0.9934). C. Evaluation of the compaction and deformation of the algal suspension of *V. carteri* spheroids by phase contrast microscopy through the determination of *V. carteri* colony circularity (N = 3, n ≥ 75, Welch *t*-test (***: p ≤ 0.001, scale bar: 250 μm). D. Observation of the surface adhesion capacity of *V. carteri* spheroids for various cell types. From left to right, phase contrast and environmental scanning electron microscopy (ESEM) observations of L929 adhered to the surface of *V. carteri* (white arrows), phase contrast observation of HDFn spheroid-like structures (black arrows), and HUVEC cell chains (red arrows) adhered to the surface of *V. carteri* microalgae. E. Evaluation of the cell adhesion-promoting properties of *V. carteri* extract. Phase contrast observation of HDFn and HUVEC cells 48 h after seeding on adhesive, anti-adhesive or *V. carteri* extract-coated surfaces (scale bar: 250 μm). PHANTAST plugin and Fiji software were used to estimate cell confluency [20], [21](n ≥ 3, Mann–Whitney *U* test (**: p ≤ 0.01)). (For interpretation of the references to color/color in this figure legend, the reader is referred to the Web version of this article.)

To build three-dimensional tissues, we relied on the compact stacking of living building blocks (LBB) formed by human cell-cellularized *V. carteri* (Fig. 3A). A saturated spheroid suspension served as the starting material to generate this stacking.

The diameter distribution of the spheroids within this suspension (Fig. 3B) shows a normal distribution centred between 200 and 250 μm, accounting for 25% of the spheroids. 70% of the spheroids ranged between 150 and 300 μm in diameter. Spheroids with a diameter under 100 μm represented only less than 3% of the spheroids. 1 mL of saturated suspension of algae containing about 75 × 10^3^ spheroids thus offers a developed surface area of about 100 cm^2^. This multiplies the available surface area for cell adhesion in LBB by about 30 times the initial insert surface of a 12-well insert.

To investigate the impact of *in vitro* culture on *V. carteri* spheroid morphology, the algal suspension was maintained in standard insert culture conditions at 37 °C and 5% CO_2_ with regular basal medium changes for 21 days (D). As shown in Fig. 3C, the algal spheroids were closely spaced from the beginning of the culture (D2), but gaps were present at their intersection. The spheroids' spacing was progressively reduced by draining the excess culture medium between D7 and D21. The spheroids gradually deformed without any rupture, adopting hexagonal to square shapes due to the mechanical constraint of the sphere's compact stacking. At 21 days of culture, a significant deformation was notable; the circularity of the algae appeared to have decreased from 0.97 at D2 to 0.92 on completion of the culture. The algal suspension thus demonstrated a high capacity for compact incremental stacking and stability *in vitro* while retaining a degree of malleability resulting from its high deformability, thus allowing the provision of a large growth surface area with full interconnectivity.

### 3.4 Characterization of the cell adhesive properties of *V. carteri*

When seeded together with fixed *V. carteri* spheroids on an anti-adhesive surface, various cell types developed differing strategies (Fig. 3D). L929 murine fibroblasts had significant cell adhesion capabilities to the algal surface. In contrast, human dermal fibroblasts (HDFn) tended to organize into spherical cell aggregates before adhering to the algal surface, without covering it. Finally, human umbilical vein endothelial cells showed an intermediate strategy, in that cells remained individualized, forming small chains with some adhering to the algal surface without completely encircling it.

To evidence the microalga's cell adhesion properties, cell adhesion assays were performed on a *V. carteri* extract-coated anti-adhesive surface, compared to non-coated anti-adhesive and cell-treated adhesive surfaces. The confluence status of HDFn and HUVEC cell types was expressed as a ratio of the culture conditions shown in Fig. 3E. A confluency drop was observed for each cell type on the anti-adhesive condition, while the raw extract pre-treated condition exhibited significantly increased confluency. More specifically, for the HDFn, the confluency decreased to 41.3 ± 8.4% in the anti-adhesive and recovered to 73.8 ± 13.8% with the algal coating. This effect was reproduced for HUVEC, increasing from 8.7 ± 3.0% to 53.9 ± 10.7% of maximal confluency. This led us to infer that some components of *V. carteri* raw extract, presumably glycoproteins and glycans, promote cell adhesion for both human mesenchymal and endothelial cells.

### 3.5 Algal ECM components prospective analysis

The Phytozome portal dedicated to plant genomic data repository and analysis has been supplemented recently with the *V. carteri* complete genomic sequences provided by Prochnik et al. [13]. A keyword-based search for ECM components performed on the *Volvox* deduced proteome data highlighted the presence of proteins that could support our observations regarding cell adhesion. Our focus was first on pherophorins, a group of proteins classified as true algae ECM components, seven of which have been identified experimentally in *V. carteri* and have equivalents in *Chlamydomonas reinhardtii*, *Gonium pectorale* and *Pandorina morum* [10]. More than 80 hypothetical protein sequences containing a pherophorin domain were identified in the *V. carteri* proteome. This very high number appears in good agreement with the status of pherophorins as main component of the Volvox ECM, which in turn forms the essential of the algae architecture. The proteins identified appeared highly heterogeneous in their sequences and did not share more than 35% identities when analyzed with each other by pairwise comparison. A lot of them exhibited as expected an amino acid content highly enriched in proline residues (between 10 and 25% on average), sometimes peaking at 40% because of the presence almost exclusive successions of prolines or iterations of motifs composed of prolines and serines only.

Besides pherophorins, other proteins likely to be involved in the composition of an extracellular matrix promoting the adhesion of exogenous cells were also found in the database. 10 potential proteins related to collagen type IV and type XIII were found encoded by the *Volvox* genome. Some but not all of them presented a high (20–30%) glycine content as well as the typical rhythmicity of G-X-Y patterns observed in collagens, suggesting a structural homology via a triple helix organization [14]. A few also presented an additional RGD motif that may serve as a binding motif for integrin-like proteins [15]. Six predicted proteins similar to one or more human laminin subunits α, β, or γ were identified as well and may also contribute to this primitive yet complex extracellular matrix within and on the colony's surface. 14 sequences apparently coding for proteins with a fasciclin domain were also found encoded by the *Volvox* genome. Alongside them, proteins involved in glycoprotein modification (e.g., exostosin 1 and 2), cell adhesion (e.g., teneurin), and protein interactions (e.g., von Willebrand factor) were also present.

### 3.6 Characterization of the stiffness of *V. carteri* spheroids

To accurately define the mechanical properties of the algal suspension at the cell level, the nanoscopic stiffness of rehydrated 70% ethanol-fixed spheroids was assessed using nanoindentation. Simultaneously, the spheroid's diameter was measured (n = 95). The load increased with indentation. The Young modulus was determined as the slope of the linear part of the curve between 1 μm and 3 μm, considering Hertz's sphere-sphere contact model. Young's moduli as a function of the diameter of the spheroids are plotted in Fig. S2. Young's moduli ranging from 1.5 to 4 kPa for diameters ranging from 100 to 400 μm were obtained. The average Young's modulus of *V. carteri* spheroids was 2.8 ± 0.5 kPa. The diameter of the spheroids did not seem to affect their mechanical properties, as no correlation was observed between *V. carteri* spheroids' Young's modulus and diameter (Pearson's correlation test; Pearson's r = 0.063).

### 3.7 In vitro self-assembly and *in vivo* biocompatibility of *V. carteri*-based living building blocks: the example of neonatal human dermal fibroblasts-based modular microtissue

Morphological, proliferation, and histological studies were performed using HDFn-seeded *V. carteri* to assess macrotissue formation and its ultimate use as a soft-tissue filler.

**Fig. 4:**
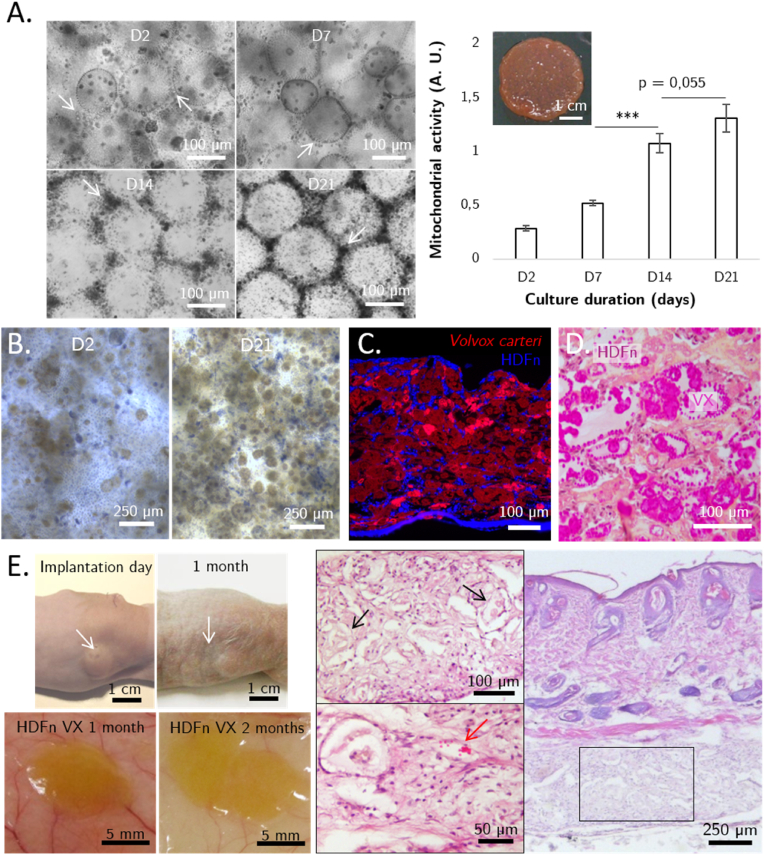
Neonatal human dermal fibroblasts (HDFn)-seeded *V. carteri* living building blocks *in vitro* self-assemble into modular macrotissue, early investigation of its *in vivo* physical stability and biocompatibility. A. HDFn proliferation and densification (pointed by white arrows) monitoring at day 2, 7, 14 and 21 (N = 3, ANOVA analysis–Post Hoc Bonfferoni test (***: p < 0,001)) provided with a photograph of the macrotissue obtained after 21 days of culture**,** B. Monitoring of alkaline phosphatase activity in HDFn seeded in the *V. carteri*-based macrotissue system along the culture duration, C. DAPI-stained HDFn (blue)-seeded Rhodamine-crosslinked *V. carteri* (red) living building blocks-based macrotissue epifluorescence observation, D. Histological analysis of HDFn-seeded Rhodamine-crosslinked *V. carteri* living building blocks-based macrotissue stained with Hematoxylin Eosine Safran. E. Post-implantation and 1-month follow-up photographs of the implant site (white arrow) are provided with macroscopic observation of *V. carteri* HDFn pseudotissue implant one month and two months post implantation, and a 1-month post-implantation histopathological examination of the implanted HDFn-seeded *V. carteri* living building blocks-based macrotissue showing signs of algal degradation (black arrow) and tissue vascularization (red arrow). (For interpretation of the references to color/color in this figure legend, the reader is referred to the Web version of this article.)

We demonstrated that HDFn adhered to the surface of the microalga in the form of sphere-shaped aggregates, whereas when grown in 2D, HDFn expands in a fusiform manner, developing focal adhesions and filopodia. HDFn mitochondrial activity was quantified using MTS testing and appeared to increase throughout the culture. This activity was multiplied by 4.5 between days 2 and 21, going from 0.29 to 1.31. This rise in activity was associated with the densification of fibroblast-based tissue structures at the periphery of the spheroids, highlighted by white arrows in Fig. 4A, demonstrating HDFn proliferation. Upon 21-day culture completion, a macrotissue with integrity, although flexible and fragile, was obtained. While cultured on the surface of *V. carteri* spheroids, HDFn also exhibited alkaline phosphatase activity starting at day 2, whereas no enzymatic activity was detected when spread onto a 2D culture surface (Fig. 4B). More and more fibroblasts were found to be positive for alkaline phosphatase expression as cells proliferated.

The fluorescent rhodamine-coupled *V. carteri* suspension showed a homogeneous distribution of fibroblasts within the whole tissue thickness (Fig. 4C). However distorted, the algal spheroids remained complete and still offered a surface on which the fibroblasts expanded. Cellular clusters were formed in the cavities resulting from the stacking of multiple LBBs. Upon HES staining, this organization was recovered, and the integrity of the algal morphology, in bright pink, was observed. Its mucosal content was discernible as a thin, pale pink-to-yellow veil. Finally, self-organized cell sheets and clusters were supported by a fibrous extracellular matrix.

To ascertain the *in vivo* behavior of the HDFn-seeded *V. carteri*-based macrotissue and the resulting body response, subcutaneous implantation in athymic mice was realized. The 3D macrotissue was collected in a syringe due to its brittleness, and a gelatinous suspension volume of about 150 mm^3^ was injected on the backs of mice, creating an externally visible protruding implant. This expanded volume, indicated by an arrow in Fig. 4E, remained detectable and unchanged in size and shape for the entire experiment duration. A bovine collagen gel injection was used as a control condition, to mimic an equivalent augmentation. Although the same material amount was initially injected, the bovine collagen control mostly disappeared, leaving only a small, barely detectable mass under the mouse skin at 1-month post-implantation (data not shown). In contrast, the HDFn-seeded *V. carteri*-based implant exhibited a large mesenchymal tissue-like structure penetrated by the surrounding blood vessels on both the 1-month and 2-month post-implantation necropsies, demonstrating the great *in vivo* stability of the remodelled tissue construct. The homogeneously cellularized fibrous tissue showed remnants of spherical structures attributable to the breakdown of *V. carteri* spheroids. Histological analyses of the injected HDFn-seeded *V. carteri* biopsy specimens showed the augmentation of this thick mesenchymal tissue and the surrounding tissues without any evidence of inflammation one month after implantation (i.e., absence of eosinophils, neutrophils, macrophages, or other remaining inflammatory cells). Thus, tissue remodelling and vascularization demonstrate unmistakable colonization of the implant's interstitial spaces subsequent to stromal growth. A non-cell-seeded *V. carteri*-based implant also developed a substantial mesenchymal tissue-like structure (Fig. 4. D.).

### 3.8 In vitro evaluation of *V*. *carteri*'s influence on stem cell fate

Both human mesenchymal and murine embryonic stem cells were seeded onto our *V. carteri*-based LBB system without any adipogenic-inducing supplementation. Cell morphology and engagement in the adipogenic pathway was monitored by staining their lipid content with Oil Red O, counterstained with hemalun, or counter-labelled with DAPI and phalloidin for the observation of their cell nucleus and actin cytoskeleton, by optic or confocal microscopy, respectively.

**Fig. 5:**
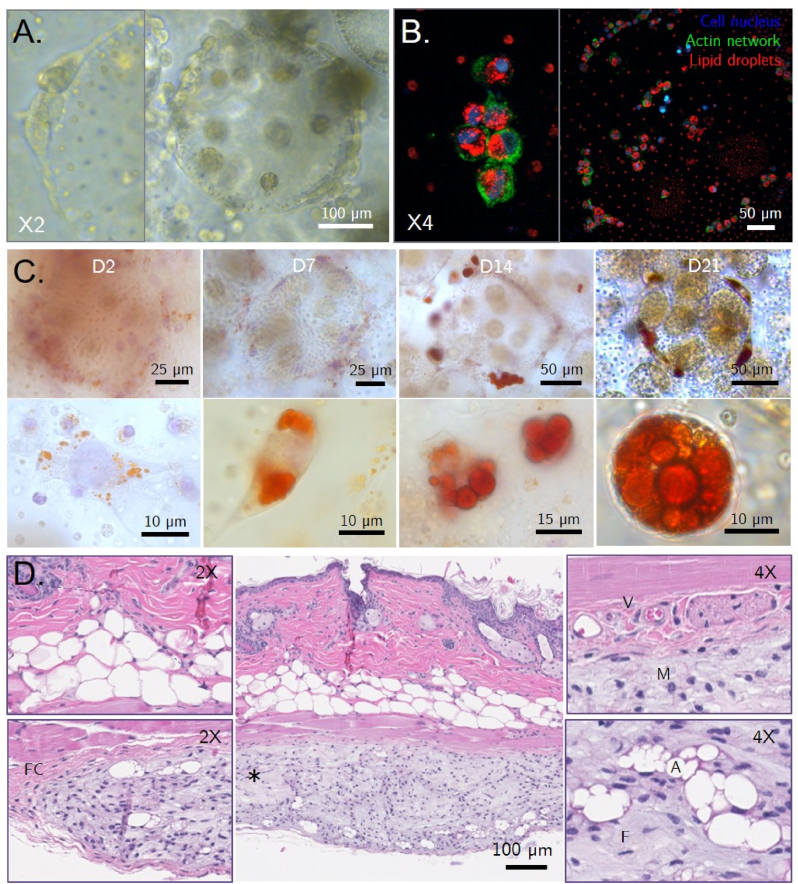
*V. carteri* demonstrates an adipogenic effect both *in vitro* and *in vivo*. A. 48 h human adipose-derived stem cells (hASC)-seeded *V. carteri* building block phase constrast observation; B. Fluorescence confocal microscopy observation of hASC-seeded *V. carteri* building blocks after 48 h, showing the adhesion of hASCs displaying adipogenic maturation signs in the production of numerous Oil Red O-stained lipid droplets; C. Monitoring of C3H/10T1/2 murine embryonic cells adipogenic differentiation upon culture in a *V. carteri* living building blocks environment, depicting both lipid droplet accumulation and expansion. D. Histopathological analysis of a 1-month post-implantation *V. carteri*-only suspension subcutaneous injection in a nude athymic mouse model demonstrates histogenesis and adipogenic effects, exhibiting the development of a mesenchymal tissue including adipocytes islets (*: injected implant, FC: fibrous capsule, V: blood vessels, M: macrophages, A: adipocytes, F: fibroblasts, purple arrow: cellular infiltration). (For interpretation of the references to color/color in this figure legend, the reader is referred to the Web version of this article.)

As seen in Fig. 5A, human adipose tissue-derived mesenchymal stem cells (hASC) were able to adhere, spread, and partially cover the surface of the algal spheroids, developing membrane protrusions similar to those exhibited when cultured in 2D. While remaining adherent and in clusters on the surface of *V. carteri* spheroids, some hASC became rounder within 48 h, diverting from the characteristic morphology of mesenchymal cells, as shown by the condensation of their actin cytoskeleton around the nucleus (Fig. 5B). The formation of dozens of lipid microdroplets of 1–3 μm in diameter, typical of adipogenic pathway drift, was observed in their cytoplasm. hASC also became polarized, drawing their nuclei closer to the cell membrane and concentrating the lipid droplets in a central position, an advanced indicator of adipocytic maturation. The roundness of the cell nucleus also seemed altered. The appearance and maintenance of micrometric lipid droplets in hASC cultured in 3D on the *V. carteri*-based LBB system for up to 21 days confirmed the adipogenic differentiation of the stem cells (Fig. S3). The size of each lipid droplet did not change considerably between D2 and D21. However, the increase in the volume of nascent preadipocytes was correlated with the increase in the number of lipid droplets, reflecting an overall increase in the lipid content of the tissues.

To ensure the replicability of the effect observed with hASCs, while broadening the pluripotency of the cells used, we aimed to apply our culture system to C3H/10T1/2 murine embryonic stem cells. C3H/10T1/2 were noted to adhere to the surface of *V. carteri* spheroids as cell aggregates (data not shown) and showed lipid microdroplet formation of about 1 μm in diameter as early as 48 h, demonstrating once again the *in vitro* adipogenic effect of the *V. carteri* substrate. While no significant increase in C3H/10T1/2 cell size was observed, their lipid droplets significantly expanded as the culture progressed. Lipid droplets with growing diameters could be identified at 7, 14, and 21 days of culture (Fig. 5C). While mainly maintaining anchorage points at the colony surface, some C3H/10T1/2 cells were found to be fully round, which, together with the presence of lipid droplets, demonstrates their adipocytic differentiation.

### 3.9 In vivo evaluation of *V. carteri*'s influence on stem cell fate

Given the efficiency of the *V. carteri* suspension to induce histogenesis and influence cellular behaviour *in vitro*, we sought to explore the potential of an injectable composed exclusively of a suspension of rehydrated 70% ethanol-fixed *V. carteri* spheroids. This suspension was injected subcutaneously into an athymic mouse model. A significant reduction in the injection volume in the first few days following implantation was observed. This reduction was mainly related to the drainage of residual fluid and the reconfiguration of the algal sphere packing, as the injectable was noted to still contain a 44% V/V buffer. The overall appearance and histopathological analysis of the integrated implant (Fig. 5D) did not show any particular dispersion of *V. carteri* spheroids throughout the tissues, as both closely spaced injection spots remained distinct throughout the implantation. When considering only the solid fraction, a 3.5- to 4-fold reduction in implant volume was noted. Yet, a remodelled translucent tissue structure remained visible, while no inflammation-related clinical signs (i.e., redness, swelling) were detected.

The anatomopathological study showed the substitution of the *V. carteri* suspension by a mesenchyme-like tissue. This 500-μm-thick remodelled tissue presented a homogeneous extracellular matrix, cellular infiltration, and integration into a fibrous capsule structurally similar to the interstitial connective tissue, overlaid with standard tissue layers of epidermis, dermis, dermal white adipose tissue, and the panniculus carnosus without visible alteration or algal infiltration. No vascular structures could be identified within the remodelled tissue. However, blood vessels were found in the surrounding interstitial tissue, strongly suggesting that the remodelled tissue may be infiltrated and supplied by non-observable capillary systems. The neoextracellular matrix showed no remaining algal structures but comprised numerous foamy macrophages and fibroblasts, indicating that material degradation and matrix remodelling could still be ongoing under mild inflammatory conditions. Interestingly, in all grafted animals, mesenchymal tissue displayed niches of about ten growing adipocytes distributed throughout the implant, particularly in its lower part.

## 4 Discussion

### 4.1 An alternative support of vegetal origin for animal cell culture

Tissue engineering strategies are generally directed toward the molecular, structural, and mechanical reproduction of tissue microenvironments *in vivo* to initiate, regulate, and guide the regeneration process [16]. Materials derived from animal ECM are generally preferred for the formation of scaffolds, in which they participate both as structural elements and as providers of an appropriate cellular niche. Yet their use still raises challenges to sustainably promote tissue regeneration [17]. This observation has prompted researchers to take other paths and explore different pre-existing systems, even those apparently distant from the ECM organization observed in animals. All these systems possess support structures likely to be tolerated more correctly because of their biological character. *V. carteri* seems to be a good candidate in this perspective. Thus, on non-adherent surfaces, the addition of a coating composed of *V. carteri* cell extract significantly increased the cell adhesion of human fibroblasts and endothelial cells. Furthermore, murine and human cells of mesenchymal and endothelial origin adhered to the surface of the preserved algae. Although the cells used adopted variable adhesion strategies, all adhered well to the proposed support, demonstrating the great potential of *V. carteri* as a cell carrier.

The exact nature of the different molecular elements that participate in the underlying architecture of the spheres of *V. carteri* is not fully elucidated and remains to be investigated. However, glycoproteins known as pherophorins (Ph) hold a special place. Pherophorins are predominant in the composition of *V. carteri*, contained and specific to each algal compartment from the cell walls, and inner membranes to ECM meshes [18,19]. Following the complete sequencing of *V. carteri* genome [13], more than 80 deduced protein sequences appeared to be related to pherophorins and/or harbour at least a pherophorin domain. However, to our knowledge, only seven pherophorins have been examined experimentally, mainly for developmental biology purposes [[20], [21], [22], [23]]. As hydroxyproline-rich glycoproteins (HRGPs) with Ser((hydroxy-)Pro)n repeats in the centred HR domains, pherophorins are classified as extensins. They also contain saccharide-binding specificities in their A and B side domains, a feature present in lectins as well [10]. These molecular specificities allow self-assembly and autocatalytic crosslinking, producing an insoluble fibrous network, as observed for the DZ1 and DZ2 pherophorins of the deep zone [20]. On account of their hydroxyproline-rich composition, fibrillar structure, and function as crosslinking and scaffolding proteins, pherophorins are considered analogous to animal collagen in the building of ECM architectures [11]. The predominance of hydroxyproline residues in their respective protein sequences largely contributes to this view. In addition to the structural maintenance of the collagen I triple helix [24,25], proline hydroxylation has been shown to be essential for the binding and stabilization of integrins, and subsequent cell adhesion of integrins [26]. Pherophorin I and II, two cellular zone pherophorins of *V. carteri*, contain 10.75% and 7.72% proline respectively, all of which are potential targets for hydroxylation. Interestingly, the SSG185 protein, also isolated from the cellular zone, harbours a very high hydroxyproline content (19.17%) and has recently been classified as a pherophorin as well [27]. Pherophorin S, the major component of the deep zone, peaks at 18.19% proline residues, most if not all being hydroxylated. In some portions of the Pherophorin S and SSG185 sequence, the hydroxyprolines are not scattered but organized into a long, nearly unbroken consecutive sequence. This characteristic is found in an exacerbated way in the DZ-HRGP protein, another protein also produced in the deep zone in response to sex pheromones and wounds, which is almost exclusively composed of hydroxyprolines [28]. This consecutive arrangement is characteristic of a very marked polyproline helix (PPII) structural organization [29]. This type of helix is considered relatively stiff [30] and very probably contributes to the overall mechanical properties of the *V. carteri* sphere (see below).

Hydroxyproline residues are the main target of O-glycosylation in plants. This is initiated by the addition of Galactose and/or Arabinose and then further extended with complex poly-Arabinose or branched Galactose chains. Serine residues adjacent to hydroxyproline glycosites may also be glycosylated [31]. Because of their high hydroxyproline content, plant HRGPs are thus highly O-glycosylated and have been considered functionally equivalent to mammalian proteoglycans such as mucins [32]. Mucins are large, heavily glycosylated proteins that are the main constituent of many mucus and play important physiological roles in cell signalling, immune response, and cell adhesion [33]. Although the O-glycosylation of microalgae proteins is still poorly deciphered [34], the glycosylation profiles of HRGPs from *V. carteri* seem quite similar to the profiles observed in plants. It is composed of long glycosylated chains, mostly made up of arabinose and galactose subunits [[20], [21], [22]] which can also include mannose and glucose sub-units, as does the ISG glycoprotein [35]. Moreover, crosslinking of saccharides with phosphodiester bridges between arabinose residues, and in a single case, the additional attachment of a highly sulphated arabinomannan have been observed [36]. The ConA lectin, which specifically binds α-mannose/α-glucose on N-glycans with little cross-reactivity [37,38], enabled us to visualize the involvement of these sugars in the general architecture and cohesion of *V. carteri*. The labelling also revealed the marked presence of highly glycosylated proteins on the surface of the algal spheres. We thus hypothesize that the glycosylated HRGPs present in the cell layer of *V. carteri* may also play a mucin-like adhesive role when they are brought into contact with cells. All our observations strongly suggest that *V. carteri*'s specific organization, based on pherophorins and other potentially associated HRGPs, promotes the structural and cell-adhesive properties of *V carteri* constitutive material.

Phytozome annotations and protein sequence comparisons using blastp enabled us also to identify other proteins which may also contribute to the cellular adhesive properties of *V. carteri*. For example, additional protein sequences were found to show direct similarities to human collagens. Moreover, six predicted proteins confirmed sequence similarities with human laminin subunits. The possible joint presence of laminin alongside pherophorins and collagen-like proteins may form a primitive yet complex extracellular matrix inside and on the surface of the colony. Although tangible evidence for the synthesis of most of these proteins remains to be identified experimentally, all of the components identified could participate to a hosting structure constitution efficiently facilitating cell implantation. This structure appears to be as effective, if not more than a matrix artificially reconstituted *in vitro* from independent elements.

### 4.2 Algal properties enabling tissue engineering and soft tissue reconstruction

The preserved algae suspension is covered with animal cells and forms stackable cellblocks. The stacking organization provides infinite malleability for multi-volume shaping. The microspheres also considerably increase the adhesion surface compared to a flat or even porous material. The variability of carrier sphere sizes allows for greater compaction, while creating niches at their intersections and an interstitial continuum, ensuring complete interconnectivity. In the case of conventional scaffolds, ensuring easy diffusion of nutrients and gases is difficult because the solid phase of the material too often acts as a barrier [39]. Throughout the compaction of the spheres and the progressive reduction of the interstitial void between them, this diffusion is significantly decreased between D0 and D2. The high cell density that results from this internal compaction leads to the progressive deformation, without immediate rupture, of the algal spheres. This remodelling is favourable to an increase in the cohesion of the tissue structure created. When implanted, seeded collagen matrices generally present a strong degradability, which affects *in vivo* stability but facilitates integration *in situ*. However, although the *in vivo* resorption rate of a collagen implant can be regulated by controlling its density and degree of intermolecular cross-linking [40], it tends to generate a foreign body response [41]. When we injected a suspension of rehydrated 70% ethanol-fixed *V. carteri* spheres without any cells seeded, we noticed an absence of dispersion of the spheres throughout the tissues. One month after injection, the spheres appeared resorbed and substituted by a mesenchyme-like tissue. The experience was reproduced using agglomerated human-cell-seeded blocks. Once this *V. carteri*-based compact tissue was obtained and subcutaneously implanted, we could observe that it remained stable, highly cellularized, and vascularized up to 2 months after implantation. Again, the alga had almost disappeared. Since no significant inflammation was observed during the degradation of *V. carteri* in different implantation conditions, the algal support may have been degraded *in vivo* by a fibroblast-dependent phagocytic mechanism related to matrix homeostatic remodelling [42]. More investigations have to be conducted to evaluate tissue ingrowth and remodelling within the implant, particularly by distinguishing the respective contribution of murine and human fibroblasts to the final tissue obtained.

Biomaterials are not only passive carriers but are also designed to utilize the mechanobiology of seeded cells to promote their differentiation, a process that is more difficult to achieve in hormone-induced 2D cultures. For example, hASCs can differentiate into neurons, adipocytes, myoblasts, or osteoblasts when grown on substrates with stiffnesses measured by their Young's modulus of 0–1, 2–4, 8–10, and >30 kPa, respectively [17,43,44]. However, in the case of adipose tissues, most engineered materials demonstrate a Young's modulus that is significantly higher (E ≈ 1 MPa) than the soft tissue they aim at reproducing (1–10 kPa). Indeed, the Young's modulus of the fibrillar proteins that often make up these materials is much greater than that of tissue component assemblies, which in turn are significantly stiffer than the elastic properties of bulk tissue samples [45]. As demonstrated in the case of obesity, ECM stiffness increases impaired adipogenesis by blunting the sensitivity to insulin and lipolytic cues, as well as the secretion of adipokines [46]. *V. carteri* provides a spherical substrate with an overall stiffness of about 3 kPa that is stable regardless of colony diameter or stage of development. The mechanical properties of the sphere suspension are therefore very homogeneous. The whole presents a stiffness close to the one observed during the transition from isolated adipocytes to adipose tissue [47,48] and fully fits within the 2–4 kPa range of estimated optimal stiffness for the mechanical induction of adipogenesis. This can be attributed to the predominance of pherophorins and other HRGPs in the ECM of *V. carteri*. Both contain a high amount of hyperglycosylated hydroxyproline. Proline hydroxylation has been shown to improve collagen elasticity [49]. The hyperglycosylation of these same residues also contributes to overall flexibility. On the other hand, the rigid modules of the polyproline helices present in some HRGPs have an antagonistic effect since they maintain a certain stiffness. It seems that the mechanical properties of the spheres of *V. carteri* result from a balance between these two phenomena. It has previously been shown that *in vitro* recreation of adipose rigidity can stimulate the adipogenesis of human adipose-derived mesenchymal stem cells without the use of chemical media additives [50], and some explanations have been proposed for this phenomenon. Mesenchymal stem cells cultured on flexible substrates significantly reduce their cell surface area and aspect ratio as a result of low cytoskeleton contractility [51], influencing adipocyte differentiation via ERK signaling [52], promoting the upregulation of gene expression of all three adipogenic markers (i.e., PPARγ, CEBPα, and aP2), and lipid accumulation [50]. The actin cytoskeleton reorganization dynamics also affect the roundness of the nucleus during adipocyte differentiation [53]. In addition to adipocyte stiffness adequacy, *V. carteri* colony sphericity may also foster adipogenesis. Indeed, the cellular geometry driven by cellular contractility influences MSC differentiation: cells with rounder shapes that promote low contractility are more likely to follow an adipocyte lineage, while cells in elongated shapes promote increased myosin contractility, which enhances pathways associated with osteogenesis [51]. The static strain of adipocytes on neighbouring preadipocytes promotes differentiation [54,55]. Regardless of its origin, *V. carteri's* mechanical mimicry with adipose tissue is able to induce adipocytic differentiation of mesenchymal stem cells very efficiently.

### 4.3 Durable, implantable, biocompatible, and non-inflammatory building blocks

The microalga was found to be physically stable under culture conditions and maintain high levels of cell viability. A balanced inflammation (i.e., not excessive in its intensity and duration) stimulates tissue renewal, allowing the formation or remodelling of tissues. The inflammatory and immunogenic potential of *V. carteri* was thus evaluated *in vitro*. Our experiments showed the absence of a global pro-inflammatory response. When Schenck et al. implanted *C. reinhardtii*, a V*. carteri*-related unicellular algae, either in athymic nude mice or zebrafish *in vivo* models, they did not observe any significant inflammatory response either [56]. The graft of what Obaíd et al. call “photosynthetic scaffolds” (that is, scaffolds loaded with *C. reinhardtii)* did not trigger any deleterious local or systemic immune responses in a 90-day follow-up, allowing full tissue regeneration in humans [57]. *V. carteri*'s anti-inflammatory effect could further be investigated as a decrease in proinflammatory IL-9 and IL-15 were observed for all algal-treated conditions. Several green algae phytochemicals such as hydroxylated fatty acids, chlorophyll-derived pheophorbides, carotenoids, and glycoglycerolipids possess anti-inflammatory effects by decreasing nitric oxide intracellular levels [58,59]. *C. debaryana*, and *P. japonica*, used as dry biomass or extracts, respectively, have demonstrated their anti-inflammatory efficacy in the treatment of intestinal mucosa inflammation *in vivo* [60,61]. Given the phylogenetic proximity of *C. reinhardtii* and *V. carteri,* this allows for great confidence in the overall biocompatibility of the algae.

When human dermal fibroblasts (HDFn) were cultivated at the surface of *V. carteri* beads, their alkaline phosphatase (AP) activity could be detected exclusively when grown in 3D. A high level of AP correlates with undifferentiated pluripotent stem cell phenotypes [62], Indeed, mechanically confined fibroblasts have shown the ability to reprogram stem cell-like cells and rejuvenate, demonstrating alkaline phosphatase activity [63]. We suspect that *V. carteri* may induce fibroblast dedifferentiation by forming fibroblast aggregates and confining them into specific niche environments. However, AP is also ubiquitous and known to be actively produced in bone, liver, and kidney [64]. Therefore, additional research, including the determination of pluripotency transcription factor expression, should be conducted on HDFn seeded on *V. carteri* to validate our statement. Moreover, because fibroblasts are contractile cells and *V. carteri* exhibits signs of deformability, these cells have been able to handle this interstitial volume drain by substituting it with extracellular matrix while proliferating, as demonstrated by the increase in mitochondrial activity and histological analysis of ECM neosynthesis. Altogether, our cellularized *V. carteri* living building blocks were found to be suitable for the construction of three-dimensional tissues showing an even distribution of cells and matrix secretion over their entire thickness.

### 4.4 Differentiation orientation towards adipose tissue

Adipose tissue engineering has recently emerged as a growing field in regenerative medicine [65]. Unfortunately, the biomaterials currently available for adipose tissue substitution still face limitations in guaranteeing long-term tissue regeneration. These materials usually generate a foreign body response, stabilizing and isolating the volume from the rest of the body via fibrous tissue encapsulation [50,66]. Recently, research efforts have focused on the design of bioactive materials that would target specific regenerative mechanisms [16]. *V. carteri* living building blocks-mediated culture generated massive lipid droplet synthesis and maintenance in culture duration on both C3H/10T1/2 and hASC, undeniably demonstrating the capabilities of the alga as a material to promote adipogenesis without any addition of induction factors. hASC exhibited both cytoskeleton anisotropy as well as nucleus polarization and roundness alteration as early as 48 h, reflecting a significant shift towards the adipogenic pathway [47,67]. By contrast, 2D adipogenic differentiation of both C3H/10T1/2 and hASC would necessitate 7–10 additional days of culture post-confluency in an adipogenic induction medium including at least insulin and thiazolidinedione-insulin sensitizers [68,69]. While the number of microdroplets per cell for hASC increased as the culture progressed, no significant growth in lipid microdroplet size was observed. On the contrary, a distinct lipid microdroplet expansion could be observed for C3H/10T1/2. This differentiation limit can be explained by interspecies differences in their lipid accumulation mechanisms, especially those regulated by PPARγ. Human adipose cells, unlike mouse adipose cells, cannot secrete endogenous PPARγ ligands *in vitro*, necessitating the addition of PPARγ agonist supplementation [70]. To achieve advanced adipocyte differentiation, *in vitro* exogenous sources of PPARγ ligands could be added by supplementation in the medium or by establishing co-culture with PPARγ ligand-secreting cells, like human adipose tissue-derived microvascular endothelial cells [71].

## 5 Conclusions

In the search for alternative tissue engineering approaches, many researchers have now overcome the daunting task of crossing the kingdoms and focusing on microalgae and plant-based materials. By taking advantage of similarities in the vascular structure of plant and animal tissues, Gershlak et al. [72] were able to use a decellularized plant tissue as a vascularized scaffold for tissue engineering applications. Moreover, the incorporation of *Spirulina* in electrospun scaffolds has demonstrated improvement in cytocompatibility by providing a wide range of vital nutrients to the cells [73]. Similarly, living photosynthetic *C. reinhardtii* volvocine algae implanted in a wound defect effectively delivered oxygen *in situ* to limit hypoxia and promote wound healing [56,57]. Here, we aimed to take advantage of *V. carteri*'s global structure and composition for soft tissue augmentation. The data presented demonstrate the potential of *V. carteri* as an innovative alternative and structuring biomaterial for soft-tissue engineering, exhibiting capabilities in sustaining cell adhesion and supporting histogenesis, both *in vitro* and *in vivo*. The 3D compact packing of algal-based cell-seeded spheroids (LBBs) offers an extensive, unique, and integrally interconnected culture surface suitable for cellularization and diffusion throughout the template. This work is a proof of concept of the feasibility of a modular approach for cell-seeded *V. carteri* living building blocks for three-dimensional tissue formation and their use for *in vivo* soft tissue regeneration. The adipogenesis-driving properties of these structures may well be directly tied to the capability of the algal support to mimic adipose tissue structurally, mechanically, and molecularly.

## Ethics approval and consent to participate

n/a.

## Consent for publication

n/a.

## Availability of data and materials

Data will be made available on request.

## Funding

MS received a fellowship from the French Ministry of Science and Technology.

## CRediT authorship contribution statement

**Mathilde Stricher:** Writing – original draft, Methodology, Investigation, Data curation, Conceptualization. **Pascale Vigneron:** Supervision, Formal analysis, Data curation, Conceptualization. **Frederic Delbecq:** Writing – original draft, Supervision, Methodology, Conceptualization. **Claude-Olivier Sarde:** Writing – original draft, Validation, Methodology, Investigation, Conceptualization. **Christophe Egles:** Writing – original draft, Validation, Supervision, Project administration, Methodology, Funding acquisition, Formal analysis, Conceptualization.

## Declaration of competing interest

The authors declare that they have no known competing financial interests or personal relationships that could have appeared to influence the work reported in this paper.

## Data Availability

Data will be made available on request.

## **List of abbreviations**

extracellular matrix: (ECM)
living building blocks: (LBB)
Dulbecco's modified eagle's medium: (DMEM)
Fetal Bovine Serum: (FBS)
Human Dermal Fibroblasts: (HDFn)
Human Umbilical Vein Endothelial Cells: (HUVEC)
Human adipose-derived mesenchymal stem cells: (hASC)
Mesenchymal Stem Cell: (MSC)
hematoxylin-eosin: (HE)
hematoxylin-eosin safran: (HES)
dexamethasone: (DEX)
isobutylmethylxanthine: (IBMX)
