# Supplementary material for: The microalga Volvox carteri as a cell supportive building block for tissue engineering
Source: Mater Today Bio. 2024 Feb 29;25:101013. doi: 10.1016/j.mtbio.2024.101013 (PMC10923841; doi:10.1016/j.mtbio.2024.101013)
Supplement: Multimedia component 1 [file mmc1.docx]

Appendix A. Supplementary data


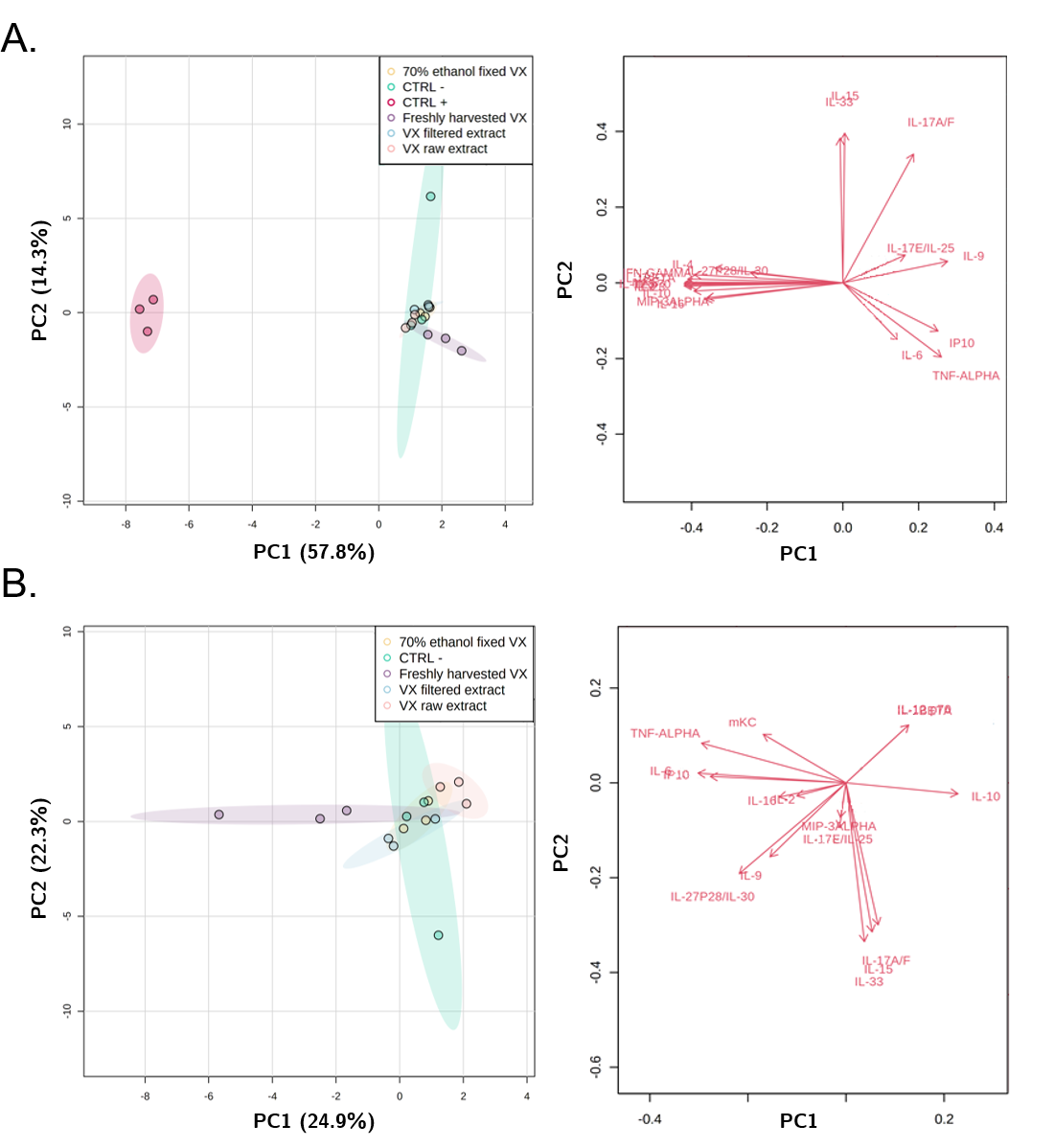


**Figure S1 - Principal component analysis (PCA) of J774.2 macrophage secretome upon 24h-exposition to *V. carteri*.** A. PCA analysis of *V. carteri*-induced macrophage secretome in regards to both untreated (CTRL-) and treated (CTRL+) controls. B. PCA analysis of *V. carteri*-induced

macrophage secretome in regards to the untreated (CTRL-) control. The supernatant cytokine

and chemokine concentration was determined by Meso Scale Discovery (N=3), processed for

normalization and PCA analysis with Metaboanalysis software [22].


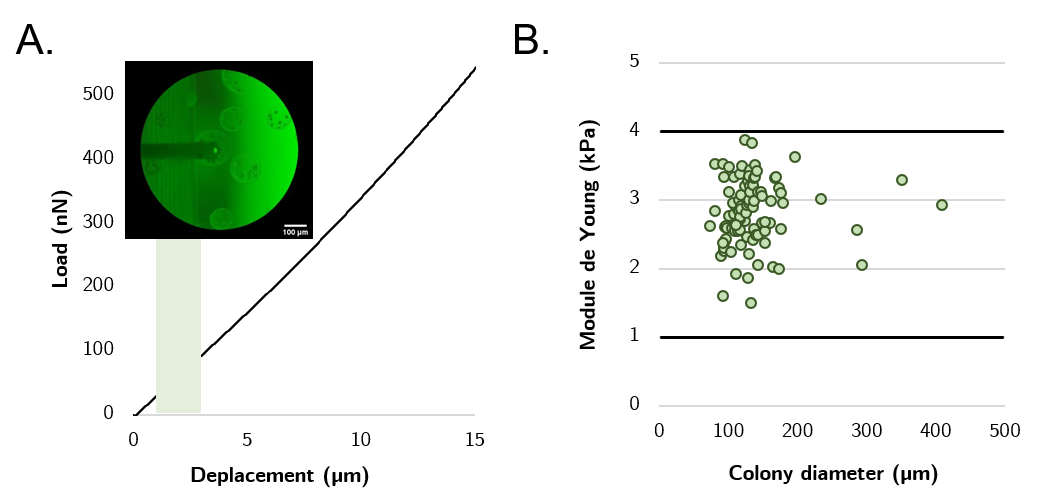
**Figure S2 - *V. carteri* stiffness determination (n=95).** A. Mean load-displacement curve obtained

upon 15,000 nm nanoindentation at a rate of 3 nm/s on a *V. carteri* colony (Probe diameter:

28 μm, stiffness: 0.48 N/m), B. Distribution of Nanoscopic Young’s modulus of *V. carteri* spheroids

as a function of their diameter (Pearson’s correlation test; Pearson’s r = 0,063).


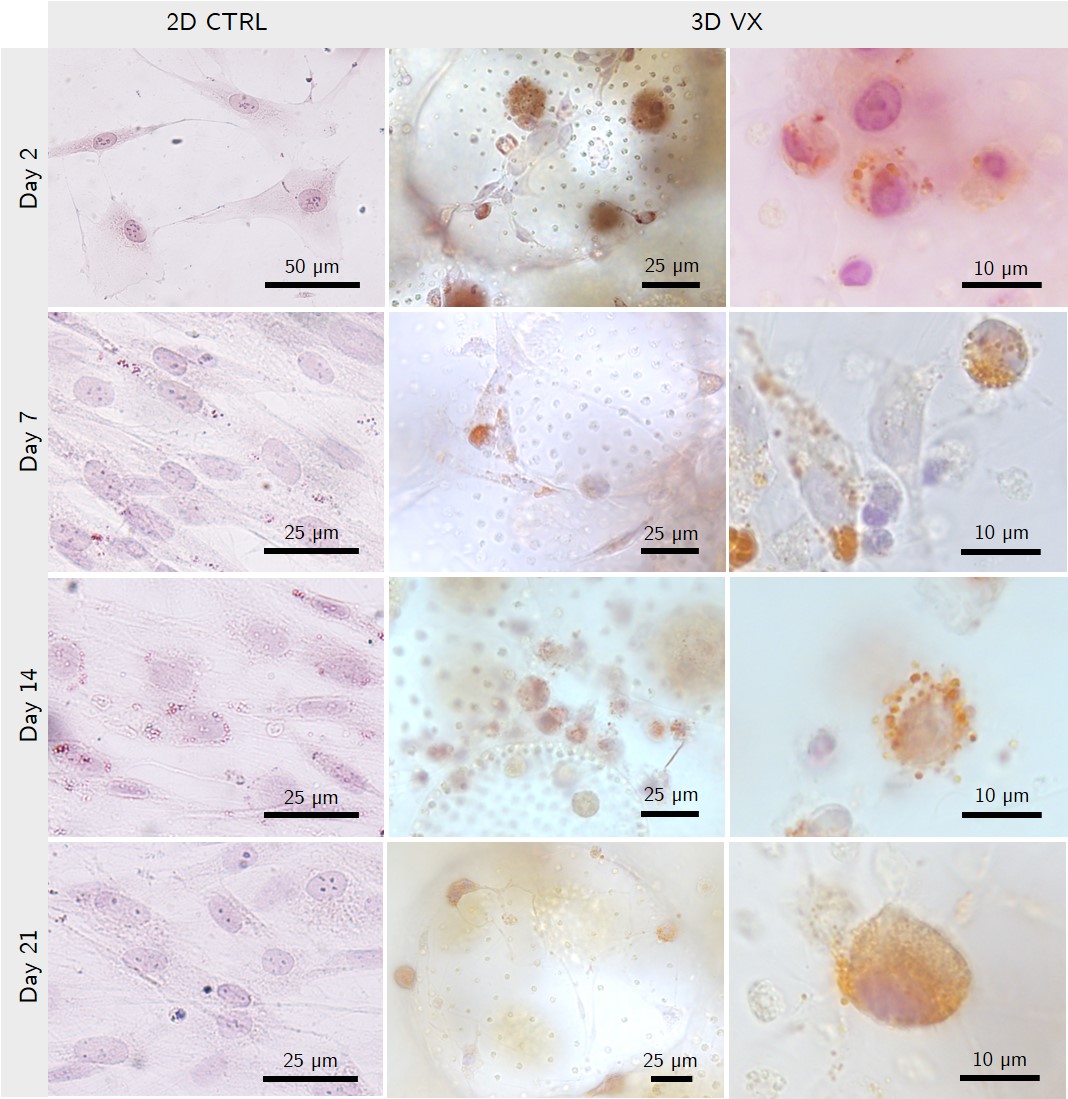


**Figure S3 - Adipose-derived stem cells adipogenic differentiation upon culture in a
*V. carteri* building blocks environment.** Bright-field microscopy observation of hASC grown
for up to 21 days using the *V. carteri* building block technique, exhibiting Oil Red O-stained lipid
microdroplets (*orange/red*) and counterstained with hemalun to reveal the cell nucleus (*purple*).
